# Immunomodulatory Effects of Poly-D,L-Lactic Acid on LL-37-Driven Rosacea-like Inflammation via Suppression of mTORC1 Signaling

**DOI:** 10.3390/ijms27146425

**Published:** 2026-07-19

**Authors:** Kyung-A Byun, Je-Young Park, Seyeon Oh, Ji Yeoun Shin, Suk Bae Seo, Kuk Hui Son, Kyunghee Byun

**Affiliations:** 1 Department of Anatomy & Cell Biology, College of Medicine, Gachon University, Incheon 21936, Republic of Korea; 2 LIBON Inc., Incheon 22006, Republic of Korea; 3 Functional Cellular Networks Laboratory, Lee Gil Ya Cancer and Diabetes Institute, Gachon University, Incheon 21999, Republic of Korea; 4 Oracle Dermatology Clinic, Seoul 06097, Republic of Korea; 5 Hev Dermatology Clinic, Seoul 06035, Republic of Korea; 6 SeoAh Song Dermatologic Clinic, Seoul 05557, Republic of Korea; 7 Department of Thoracic and Cardiovascular Surgery, Gachon University Gil Medical Center, Gachon University, Incheon 21565, Republic of Korea; 8 Department of Health Sciences and Technology, Gachon Advanced Institute for Health & Sciences and Technology (GAIHST), Gachon University, Incheon 21999, Republic of Korea

**Keywords:** LL-37, mechanistic target of rapamycin complex 1, poly-D,L-lactic acid

## Abstract

Rosacea is a chronic inflammatory skin disorder driven by dysregulated cathelicidin processing and excessive LL-37, which triggers a circuit involving Toll-like receptor 2 (TLR2)/kallikrein-5 (KLK5)-dependent amplification and downstream mechanistic target of rapamycin complex 1 (mTORC1), NF-κB, and NLR family pyrin domain containing 3 (NLRP3) inflammasome pathways. We hypothesized that poly-D,L-lactic acid (PDLLA) could attenuate this inflammatory cascade by inducing macrophage-derived interleukin (IL)-10. PDLLA increased IL-10 secretion from THP-1-derived macrophages in a dose-dependent manner. In LL-37-stimulated HaCaT keratinocytes, LL-37 decreased phosphorylated signal transducer and activator of transcription (pSTAT3)/STAT3, DNA damage-inducible transcript 4 (DDIT4), and phosphorylated AMP-activated protein kinase (pAMPK)/AMPK while increasing phosphorylated protein kinase B (pAKT)/AKT and mTORC1 activation; conditioned media from PDLLA-treated macrophages (CM_PDLLA_) restored pSTAT3/STAT3, DDIT4, and pAMPK/AMPK, reduced pAKT/AKT, and suppressed pmTOR/mTOR. CM_PDLLA_ attenuated downstream inflammatory responses, including NF-κB nuclear translocation, VEGF production, and NLRP3-inflammasome-mediated IL-18 secretion. These findings were validated using an intradermal LL-37-injected mouse model. Compared with the normal control/saline group, LL-37/saline decreased IL-10, pSTAT3/STAT3, DDIT4, and pAMPK/AMPK while increasing pAKT/AKT, pmTOR/mTOR, pS6K/S6K, TLR2/KLK5/LL-37, NF-κB, VEGF, and NLRP3 inflammasome/IL-18 signaling; PDLLA partially restored the STAT3/DDIT4–AMPK regulatory pattern and suppressed these disease-associated signals. Consequently, PDLLA treatment led to a pronounced reduction in clinical lesion area. Overall, PDLLA may attenuate LL-37-driven cutaneous inflammation by promoting an IL-10-linked STAT3/DDIT4–AKT/AMPK program that suppresses mTORC1 and disrupts cathelicidin amplification, supporting its potential as an injectable immunomodulatory approach for rosacea-like skin inflammation.

## 1. Introduction

Cathelicidin is a key component of cutaneous innate immunity. In human skin, the cathelicidin precursor (human cationic antimicrobial protein 18 [hCAP18]/cathelicidin antimicrobial peptide [CAMP]) is proteolytically processed into active peptides, including LL-37, which contribute to antimicrobial defense and barrier homeostasis [1]. However, dysregulated cathelicidin processing and excessive LL-37 can amplify skin inflammation; they are strongly implicated in inflammatory dermatoses with prominent neurovascular and innate immune features, such as rosacea [1,2]. A mechanistic axis in LL-37-driven cutaneous inflammation involves Toll-like receptor 2 (TLR2) and epidermal serine proteases. In rosacea skin, increased TLR2 expression promotes the release and activity of kallikrein-5 (KLK5), a critical protease that enhances cathelicidin processing and increases the abundance of inflammatory LL-37 peptides [2,3]. These interactions create an epidermal amplification loop in which keratinocytes serve as both a source and a target of innate inflammatory signals [3].

Downstream of excessive LL-37, multiple inflammatory programs are engaged, including NF-κB activation, production of angiogenic factors such as vascular endothelial growth factor (VEGF), and inflammasome signaling [4,5]. Notably, LL-37 can drive rosacea-like skin inflammation in vivo through NLR family pyrin domain containing 3 (NLRP3) inflammasome activation, providing a mechanistic link to interleukin (IL)-1 family cytokine maturation and the amplification of tissue inflammation [4].

Recent evidence suggests that mechanistic target of rapamycin complex 1 (mTORC1) signaling is a primary driver of LL-37-mediated skin inflammation. In rosacea, hyperactive mTORC1 signaling establishes a positive feedback loop with cathelicidin [6]. Specifically, LL-37 triggers phosphorylation of mTOR at Ser2448 via TLR2, which subsequently induces the phosphorylation of ribosomal protein S6 at Ser235/236 [6]. The mTORC1 pathway activation upregulates KLK5 and cathelicidin expression, creating a self-amplifying circuit that contributes to the relapsing nature of the disease [6]. Inhibition of mTORC1 with rapamycin effectively suppresses rosacea-like inflammation in experimental models, identifying the mTORC1/cathelicidin axis as a critical control point in this inflammatory pathway [6].

Resolution of cutaneous inflammation also depends on counter-regulatory immune programs, among which IL-10 is a central anti-inflammatory cytokine. IL-10 signals through the IL-10 receptor to activate Janus kinase (JAK)–signal transducer and activator of transcription (STAT) pathways; this signaling is required for canonical anti-inflammatory actions in macrophages [7]. These IL-10/STAT3 programs suppress broad inflammatory gene expression and help to terminate innate immune activation [7].

In addition to the direct suppression of inflammatory gene expression, IL-10 serves as a critical molecular brake on mTORC1 hyperactivation via STAT3-dependent induction of DNA damage-inducible transcript 4 (DDIT4). Upon activation of the IL-10 receptor, phosphorylated STAT3 upregulates DDIT4 expression. DDIT4 is a potent endogenous inhibitor of mTORC1 that effectively decouples inflammatory stimuli from the mTORC1-driven amplification loop [8].

In conjunction with this transcriptional regulation, IL-10 limits mTORC1 activity by modulating upstream kinase signaling cascades. Specifically, IL-10 inhibits the phosphorylation of protein kinase B (Akt) and its downstream targets, thereby removing a key activating input for mTORC1 [9,10,11]. Simultaneously, IL-10 promotes the activation of AMP-activated protein kinase (AMPK), a central energy sensor that functions as a robust negative regulator of mTORC1 [9,10,11].

Poly-D,L-lactic acid (PDLLA) is a biodegradable polymer widely used in dermatologic and aesthetic medicine. Beyond volumization, emerging evidence suggests that PDLLA can modulate innate immune cells, promoting macrophage phenotypes associated with tissue repair and increased IL-10 expression [12].

Considering the regulatory role of IL-10 in limiting mTORC1 hyperactivation, we hypothesized that PDLLA could attenuate LL-37-induced skin inflammation by upregulating IL-10 expression. We propose that PDLLA-induced IL-10 interrupts the mTORC1/cathelicidin feedback loop in keratinocytes through a multi-pronged mechanism that activates the IL-10–STAT3–DDIT4 axis to inhibit mTORC1, suppresses Akt phosphorylation, and activates AMPK to further limit mTOR activity. The resulting reduction in cathelicidin is expected to decrease NF-κB activity and NLRP3 inflammasome activation, thereby mitigating skin inflammation.

To validate this hypothesis, we established an in vitro model using LL-37-treated human keratinocytes to determine whether PDLLA can reverse mTORC1-mediated inflammatory signaling. Subsequently, we analyzed an in vivo mouse model of rosacea-like inflammation induced by intradermal LL-37 injection.

## 2. Results

### 2.1. PDLLA-Macrophage Conditioned Media Increased STAT3/DDIT4 and AMPK While Decreasing AKT in LL-37-Treated Keratinocytes

To determine whether PDLLA promotes IL-10 production in macrophages, THP-1 cells were differentiated into macrophages using phorbol 12-myristate 13-acetate (PMA), then treated with phosphate-buffered saline (PBS) or PDLLA. PDLLA increased IL-10 secretion in a concentration-dependent manner from 50 to 200 μg/mL, with an apparent plateau at ≥200 μg/mL. Based on these findings, 200 μg/mL was selected as the optimal concentration of PDLLA for subsequent investigations (Figure S1).

To further address dose-dependent effects on mTOR-regulatory signaling, we generated conditioned media from macrophages treated with 100, 200, or 300 μg/mL PDLLA and applied these media to LL-37-stimulated HaCaT cells. The PDLLA–macrophage conditioned media (CM_PDLLA_) increased STAT3 phosphorylation at Tyr705 (pSTAT3/STAT3), DDIT4, and AMPK phosphorylation at Thr172 (pAMPK/AMPK) while reducing AKT phosphorylation at Ser473 (pAKT/AKT) and mTOR phosphorylation at Ser2448 (pmTOR/mTOR) in a concentration-dependent manner beyond 200 μg/mL. These data further support the use of 200 μg/mL PDLLA for subsequent CM-based experiments (Figure S2).

**Figure 1:**
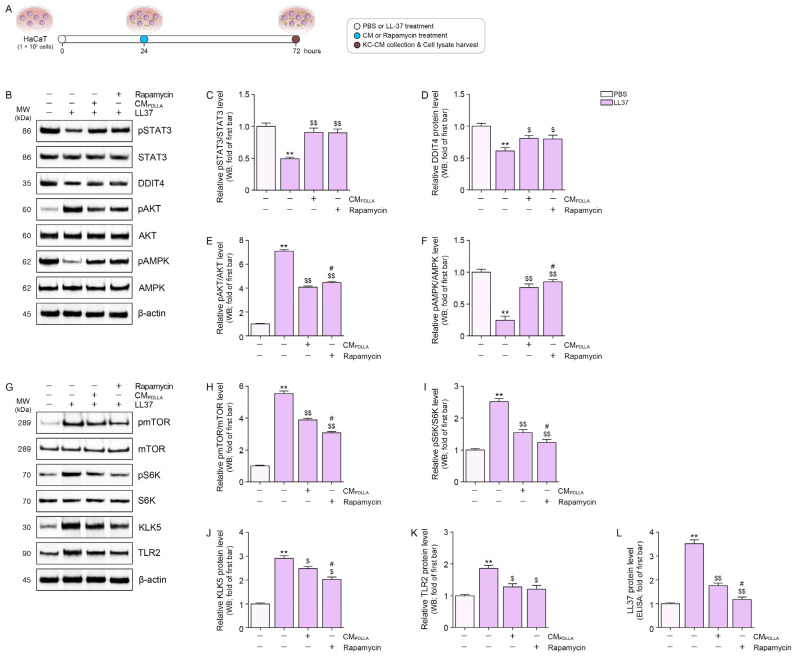
PDLLA-conditioned medium suppresses the LL-37–mTOR positive feedback loop in keratinocytes. (**A**) Experimental scheme for PDLLA-conditioned medium (CM) treatment in keratinocytes. HaCaT cells were seeded and treated with PBS or LL-37 (8 μM) for 24 h to induce inflammation. Cells were subsequently treated with PDLLA-conditioned medium (CM_PDLLA_) or rapamycin as an mTOR inhibitor control. (**B**) Western blot analysis of mTOR-related signaling molecules, including phosphorylated STAT3 (pSTAT3 Tyr705), DDIT4, phosphorylated AKT (pAKT Ser473), and phosphorylated AMPK (pAMPK Thr172). (**C**–**F**) Quantification of Western blot band intensities normalized to total protein levels. (**G**) Western blot analysis of downstream mTOR signaling molecules, including phosphorylated mTOR (pmTOR Ser2448), phosphorylated S6 kinase (pS6K Thr389), KLK5, and TLR2. (**H**–**K**) Quantitative densitometric analysis of Western blot results. (**L**) ELISA analysis of cell lysate LL-37. Overall, CM_PDLLA_ inhibited LL-37-induced mTOR activation and suppressed the LL-37–mTOR positive feedback pathway in keratinocytes. Data are presented as mean ± SD (*n* = 5 independent biological replicates). **, *p* < 0.01, vs. first bar; $, *p* < 0.05 and $$, *p* < 0.01, vs. second bar; #, *p* < 0.05, vs. third bar. AMPK, AMP-activated protein kinase; DDIT4, DNA-damage-inducible transcript 4; KLK5, Kallikrein-related peptidase 5; mTOR, mechanistic target of rapamycin; PDLLA, poly-D,L-lactic acid; SD, standard deviation; STAT3, signal transducer and activator of transcription 3; TLR2, Toll-like receptor 2.

To model LL-37-driven keratinocyte inflammation and evaluate macrophage-derived paracrine effects, HaCaT cells were treated with PBS or LL-37. After 24 h, cells received either CM_PDLLA_ or rapamycin (mTOR inhibitor) (Figure 1A). LL-37 stimulation reduced pSTAT3/STAT3 relative to the PBS control, whereas CM_PDLLA_ increased pSTAT3/STAT3; rapamycin produced a similar increase (Figure 1B,C).

LL-37 treatment decreased the DDIT4 protein levels, whereas CM_PDLLA_ increased DDIT4 relative to LL-37 alone (Figure 1B,D). Rapamycin produced a similar increase. Moreover, LL-37 treatment increased pAKT/AKT and reduced pAMPK/AMPK. CM_PDLLA_ attenuated the LL-37-induced increase in pAKT/AKT and increased pAMPK/AMPK compared with LL-37 alone (Figure 1B,E,F).

### 2.2. CM_PDLLA_ Suppressed LL-37-Induced mTORC1 Activation and Reduced Components of the LL-37 Amplification Loop

We next assessed whether CM_PDLLA_ modulates mTORC1 activation and upstream amplifiers of LL-37 production in keratinocytes. LL-37 increased the phosphorylation of mTOR (pmTOR) and S6K (pS6K), indicating the activation of mTORC1. CM_PDLLA_ reduced both pmTOR/mTOR and pS6K/S6K compared with LL-37 alone, whereas rapamycin showed stronger suppression than CM_PDLLA_ (Figure 1G–I).

Because the TLR2–KLK5 axis is central to inflammatory cathelicidin processing, we quantified KLK5 and TLR2 expression patterns. LL-37 increased KLK5 and TLR2 protein levels, whereas CM_PDLLA_ decreased both targets relative to LL-37 alone (Figure 1G,J,K). ELISA analysis of cell lysates showed reduced detectable LL-37 in the CM_PDLLA_ group compared with LL-37 alone (Figure 1L). These findings indicate that CM_PDLLA_ suppresses the TLR2/KLK5-associated positive feedback loop. Rapamycin, a potent mTORC1 inhibitor, produced stronger inhibition, suggesting that CM_PDLLA_ both inhibits mTORC1 signaling and attenuates the LL-37 amplification circuit.

To test whether STAT3 and AMPK activity are functionally required for CM_PDLLA_-mediated suppression of mTORC1, we performed additional loss-of-function experiments using a Stattic (STAT3 inhibitor) and a Compound C (AMPK inhibitor). In LL-37-stimulated HaCaT cells, Stattic reduced CM_PDLLA_-induced STAT3 phosphorylation and reversed the inhibitory effect of CM_PDLLA_ on pmTOR/mTOR. Similarly, Compound C decreased AMPK phosphorylation and restored pmTOR/mTOR despite CM_PDLLA_ treatment. These findings support the involvement of both STAT3 and AMPK signaling in the CM_PDLLA_-mediated downregulation of mTORC1 (Figure S3).

### 2.3. CM_PDLLA_ Reduced LL-37-Induced NF-κB Activation, VEGF Production, Inflammasome Signaling, and IL-18 Secretion in Keratinocytes

**Figure 2:**
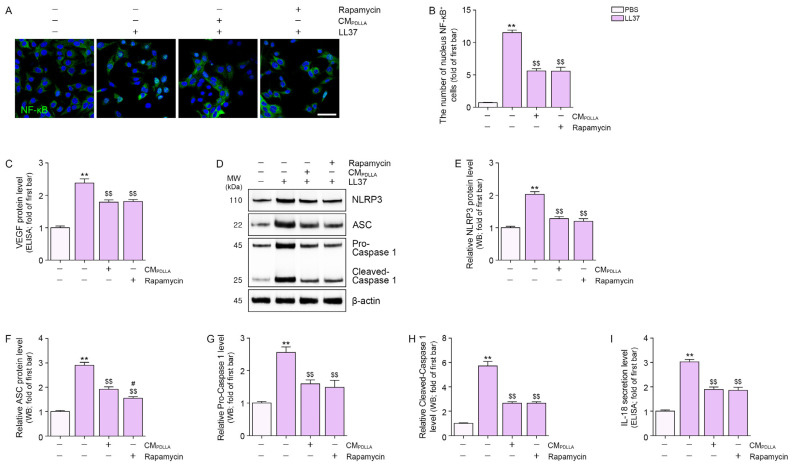
PDLLA suppresses NF-κB activation, VEGF production, and inflammasome signaling in LL-37-stimulated keratinocytes. (**A**,**B**) Immunocytochemistry (ICC) images showing nuclear translocation of NF-κB in LL-37-treated HaCaT cells. Scale bar = 50 μm. NF-κB nuclear localization increased after LL-37 treatment and was suppressed by PDLLA-conditioned medium or rapamycin. (**C**) VEGF secretion, as measured by ELISA. (**D**) Western blot analysis of NLRP3 inflammasome signaling. (**E**–**H**) Quantification of Western blot signals normalized to β-actin. (**I**) IL-18 secretion, as measured by ELISA. These results indicate that PDLLA suppresses inflammatory signaling pathways associated with LL-37-induced skin inflammation. Data are presented as mean ± SD (*n* = 5 independent biological replicates). **, *p* < 0.01, vs. first bar; $$, *p* < 0.01, vs. second bar; #, *p* < 0.05, vs. third bar. ASC, Apoptosis-associated speck-like protein containing a CARD; NF-κB, NF kappa B; NLRP3, NOD-like receptor pyrin domain-containing protein 3; PDLLA, poly-D,L-lactic acid; SD, standard deviation; VEGF, vascular endothelial growth factor.

To determine whether CM_PDLLA_ suppresses canonical inflammatory responses downstream of LL-37, we assessed NF-κB nuclear translocation, VEGF secretion, and inflammasome activation in HaCaT cells. LL-37 increased the fraction of NF-κB nuclear-positive cells, whereas CM_PDLLA_ reduced LL-37-induced NF-κB nuclear localization; rapamycin similarly reduced NF-κB activation (Figure 2A,B). LL-37 also increased VEGF secretion, which was decreased by both CM_PDLLA_ and rapamycin (Figure 2C).

Given the established role of inflammasome pathways in LL-37-driven skin inflammation, we measured NLRP3 inflammasome components (NLRP3, ASC, and pro-caspase-1) and downstream activation markers by immunoblotting. LL-37 increased the levels of NLRP3 and apoptosis-associated speck-like protein containing a CARD (ASC) while enhancing caspase-1 cleavage, consistent with inflammasome activation. CM_PDLLA_ reduced the NLRP3 and ASC expression levels and decreased caspase-1 relative to LL-37 alone; rapamycin showed a similar suppressive pattern (Figure 2D–H). Consistent with reduced inflammasome activity, IL-18 secretion was increased by LL-37 but decreased in the CM_PDLLA_ and rapamycin groups (Figure 2I).

### 2.4. PDLLA Increased IL-10 and Reprogrammed mTOR-Regulatory Signaling in an LL-37-Injection Mouse Model

**Figure 3:**
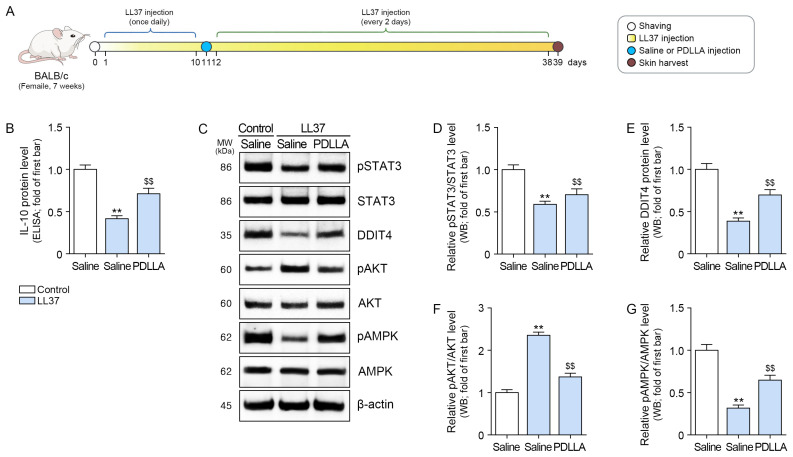
PDLLA modulates IL-10 production and mTOR-regulatory signaling in an LL-37-induced mouse skin inflammation model. (**A**) Experimental design of the LL-37-induced mouse skin inflammation model. Female BALB/c mice (7 weeks old) were divided into control/saline, LL-37/saline, and LL-37/PDLLA groups. The LL-37/saline and LL-37/PDLLA groups received daily intradermal injections of LL-37 (320 μM, 40 μL) for 10 days to induce skin inflammation whereas the control/saline group received saline without LL-37 stimulation. On day 11, mice were injected with saline or PDLLA. To prevent spontaneous healing, LL-37 was administered every 2 days thereafter. (**B**) IL-10 levels, as measured by ELISA. (**C**) Western blot analysis of mTOR-regulatory signaling proteins, including pSTAT3/STAT3, DDIT4, pAKT/AKT, and pAMPK/AMPK. (**D**–**G**) Quantitative analysis of Western blot band intensities. PDLLA increased IL-10 signaling and inhibited mTOR activation in inflamed skin tissue. Data are presented as mean ± SD (*n* = 5 mice per group). **, *p* < 0.01, vs. control/saline; $$, *p* < 0.01 vs. LL-37/saline. AMPK, AMP-activated protein kinase; DDIT4, DNA-damage-inducible transcript 4; PDLLA, poly-D,L-lactic acid; SD, standard deviation; STAT3, signal transducer and activator of transcription 3.

We next evaluated the effects of PDLLA in vivo using an LL-37-induced skin inflammation model (Figure 3A). Compared with control/saline mice, LL-37/saline mice showed reduced skin IL-10 levels. PDLLA treatment restored IL-10 toward the normal-control level (Figure 3B). In tissue lysates, LL-37/saline decreased pSTAT3/STAT3, DDIT4, and pAMPK/AMPK and increased pAKT/AKT relative to control/saline. In contrast, PDLLA increased pSTAT3/STAT3, DDIT4, and pAMPK/AMPK while reducing pAKT/AKT compared with LL-37/saline (Figure 3C–G).

Because our in vitro model suggested macrophage-mediated immunomodulation, we evaluated macrophage polarization markers in mouse skin tissues. Compared with the control/saline group, LL-37 increased the M1-associated marker CD86 and decreased the M2-associated marker CD206. PDLLA treatment reduced CD86 and partially restored CD206 in LL-37-injected skin (Figure S4A–C).

### 2.5. PDLLA Suppressed LL-37–mTOR Positive Feedback, NF-κB Activation, VEGF Production, and NLRP3 Inflammasome Signaling In Vivo

**Figure 4:**
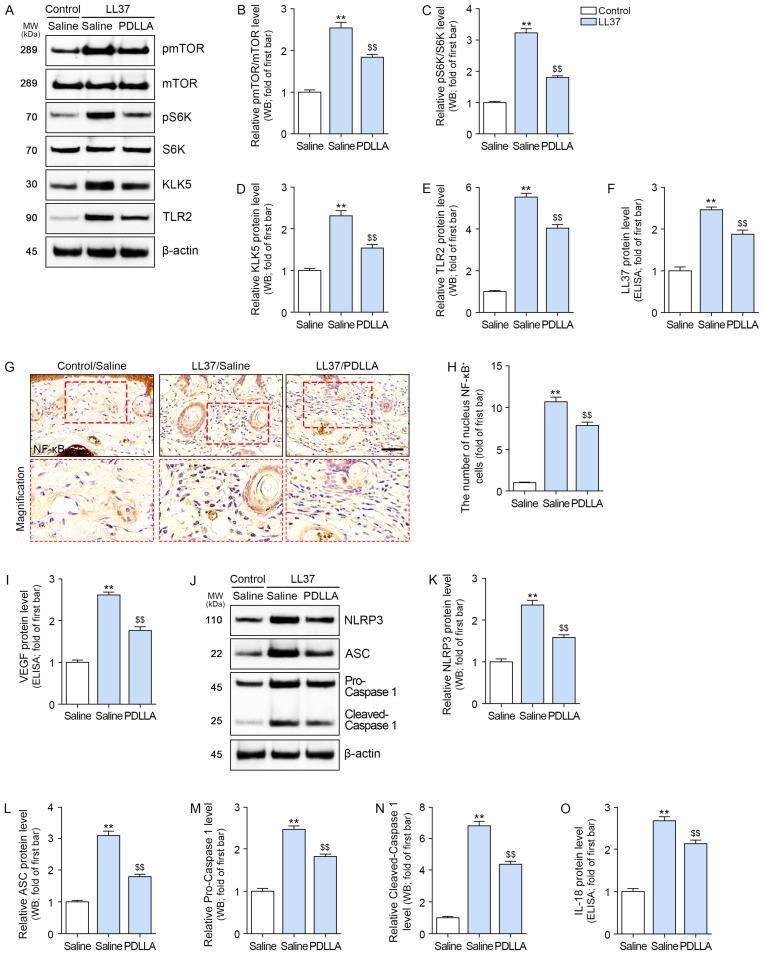
PDLLA suppresses the LL-37–mTOR positive feedback loop and inflammasome activation in vivo. (**A**) Western blot analysis of mTOR signaling and LL-37-related inflammatory proteins, including pmTOR/mTOR, pS6K/S6K, KLK5, and TLR2. (**B**–**E**) Quantification of protein expression levels. (**F**) LL-37 levels, as measured by ELISA. (**G**,**H**) NF-κB activation, as determined by immunocytochemistry. Scale bar = 50 μm. The red dashed boxes indicate the regions shown at higher magnification in the corresponding enlarged images. (**I**) VEGF levels, as measured by ELISA. (**J**) Western blot analysis of NLRP3 inflammasome signaling. (**K**–**N**) Quantification of western blot results. (**O**) IL-18 levels, as measured by ELISA. These results indicate that PDLLA suppresses the LL-37–mTOR inflammatory feedback loop and inflammasome signaling in the mouse skin inflammation model. Data are presented as mean ± SD (*n* = 5 mice per group). **, *p* < 0.01, vs. control/saline; $$, *p* < 0.01 vs. LL-37/saline. ASC, apoptosis-associated speck-like protein containing a CARD; KLK5, kallikrein-related peptidase 5; mTOR, mechanistic target of rapamycin; NF-κB, NF kappa B; NLRP3, NOD-like receptor pyrin domain-containing protein 3; PDLLA, poly-D,L-lactic acid; SD, standard deviation; TLR2, Toll-like receptor 2; VEGF, vascular endothelial growth factor.

Subsequently, we assessed whether PDLLA reduces mTORC1 activation, LL-37 amplification, NF-κB activation, and NLRP3 inflammasome signaling in vivo. Compared with control/saline mice, LL-37/saline mice showed marked increases in pmTOR/mTOR and pS6K/S6K, together with increased KLK5, TLR2, and tissue LL-37 levels. PDLLA significantly reduced pmTOR/mTOR, pS6K/S6K, KLK5, TLR2, and LL-37 compared with LL-37/saline (Figure 4A–F). In parallel, LL-37/saline increased NF-κB activation, VEGF levels, NLRP3, ASC, pro-caspase-1, cleaved caspase-1, and IL-18 relative to control/saline, whereas PDLLA reduced these inflammatory and inflammasome-associated signals compared with LL-37/saline (Figure 4G–O).

### 2.6. PDLLA Improved Tissue Inflammation, Preserved Vascular Structural Integrity, and Reduced Lesion Severity

**Figure 5:**
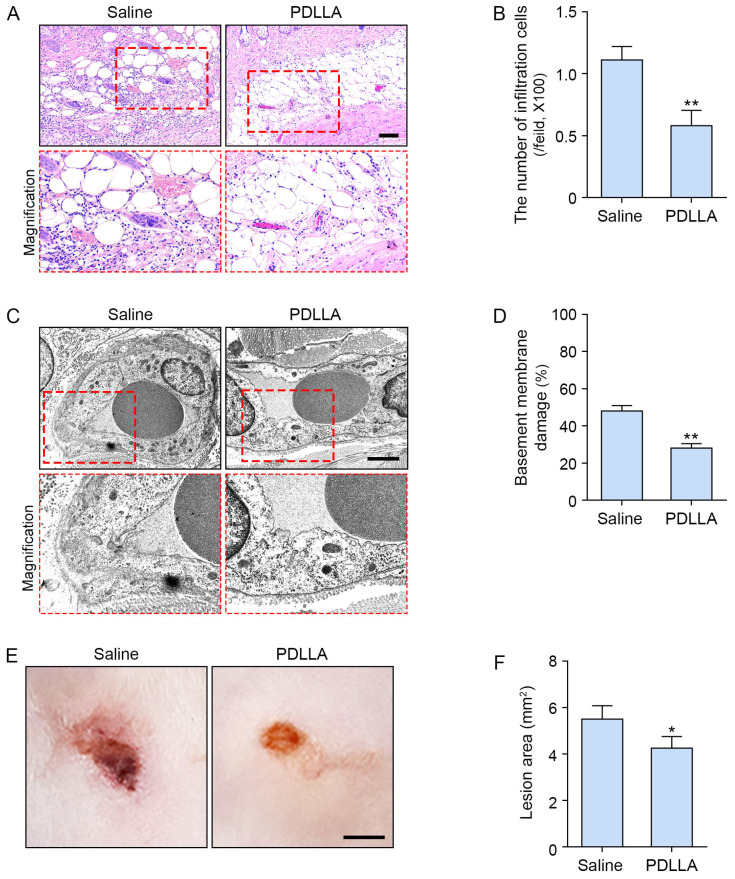
PDLLA improves vascular integrity and reduces inflammatory responses in LL-37-induced skin inflammation. (**A**,**B**) Hematoxylin and eosin staining showing inflammatory cell infiltration in skin tissue. Scale bar = 100 μm. (**C**,**D**) Transmission electron microscopy images showing vascular barrier disruption in inflamed skin and restoration after PDLLA treatment. Scale bar = 2 μm. The red dashed boxes indicate the regions shown at higher magnification in the corresponding enlarged images. (**E**,**F**) Representative images showing the macroscopic appearance of skin inflammation. Scale bar = 2 mm. PDLLA treatment reduced inflammatory cell infiltration, improved vascular barrier integrity, and attenuated visible skin inflammation. Data are presented as mean ± SD (*n* = 5 mice per group). *, *p* < 0.05 and **, *p* < 0.01, vs. saline. PDLLA, poly-D,L-lactic acid; SD, standard deviation.

Finally, we assessed whether molecular suppression translated into histopathologic and macroscopic improvement. Hematoxylin and eosin staining demonstrated reduced inflammatory cell infiltration in PDLLA-treated skin compared with the saline controls (Figure 5A,B). Transmission electron microscopy showed that LL-37-associated vascular basement membrane damage was reduced in the PDLLA group (Figure 5C,D). Gross examination of skin lesions revealed smaller and less severe lesions in PDLLA-treated animals (Figure 5E); quantitative lesion area analysis confirmed a reduction in lesion size compared with the saline controls (Figure 5F).

Representative gross images were additionally collected at day 11 (immediately after material injection), day 25 (2 weeks after injection), and day 39 (4 weeks after injection). These time-course images showed persistent LL-37-induced lesion activity in saline-treated mice and progressive improvement after a single PDLLA injection, supporting the sustained local effect of PDLLA in this model (Figure S4D).

## 3. Discussion

Rosacea is a common, chronic inflammatory dermatosis with substantial public health relevance. The estimated global prevalence of rosacea in adults is approximately 5.46% [13]. The clinical presentation of rosacea comprises heterogeneous phenotypes, including erythema/flushing, papules/pustules, telangiectasia, phymatous changes, and ocular involvement [14]. In addition to visible skin manifestations, rosacea is consistently associated with reduced quality of life, psychosocial distress, and persistent symptom fluctuation. Current therapies are typically suppressive rather than curative, and relapse is common when triggers persist or treatment is discontinued [15,16].

Rosacea is a multifactorial inflammatory disorder characterized by a complex interplay among dysregulated innate immunity, neurovascular hyperreactivity, and epidermal barrier dysfunction [16,17]. A central mechanistic hallmark of the disease is aberrant processing of cathelicidin, driven by elevated TLR2 signaling and increased activity of epidermal proteases such as KLK5. This pathological environment promotes the excessive cleavage of cathelicidin into active LL-37 peptides, which display potent pro-inflammatory and vasoactive properties [2]. This innate immune amplification promotes cytokine and chemokine production, inflammatory cell recruitment, and convergence on broader metabolic and inflammatory regulators that sustain chronic inflammation [6,18].

A key interpretive insight from our study is that PDLLA may exert anti-inflammatory effects by modulating keratinocyte signaling in an IL-10–STAT3-dependent manner, thus contributing to the suppression of mTORC1. The canonical anti-inflammatory action of IL-10 relies on IL-10-receptor-mediated activation of the JAK1/STAT3 pathway, which is essential to limit pro-inflammatory cytokine production [7].

In the present study, PDLLA-treated macrophages secreted IL-10 in a dose-dependent manner. In an LL-37-treated keratinocyte model, conditioned media from PDLLA-treated macrophages (CM_PDLLA_) significantly increased the levels of phosphorylated STAT3 and its downstream target DDIT4. These changes were accompanied by decreased Akt phosphorylation and increased AMPK activation, forming an inhibitory network that limits mTORC1 activity.

Mechanistic analyses have shown that DDIT4 is a potent negative regulator of mTORC1 with the capacity to repress upstream Akt signaling via protein-phosphatase-2A-dependent dephosphorylation [9], providing a plausible explanation for the reduced Akt/mTORC1 activity observed in the present study. Furthermore, AMPK acts as a metabolic checkpoint that inhibits mTORC1 through the phosphorylation of raptor, linking cellular energy status to inflammatory signaling [11]. Collectively, the observed signaling pattern—characterized by increased STAT3/DDIT4 expression together with reduced Akt activity and increased AMPK activation—is mechanistically consistent with the suppression of mTORC1. The finding that rapamycin mimics these effects further suggests that the LL-37-induced inflammatory phenotype is mTORC1-dependent.

Previous studies have reported that STAT3 signaling is activated in rosacea lesions. In particular, Wang et al. identified STAT3 as a key hub linking skin barrier dysfunction to rosacea aggravation and showed that barrier disruption increased STAT3 expression and immune infiltration in an LL-37-induced rosacea-like model [19]. More recently, Meng et al. demonstrated that STAT3 signaling is hyperactivated in rosacea lesions and promotes keratinocyte-driven inflammation through direct transcriptional regulation of IL-36G; inhibition of STAT3, or IL-36γ signaling attenuated rosacea-like inflammation [20]. Therefore, our data should not be interpreted as indicating that global STAT3 activation is uniformly protective in rosacea. Rather, the apparent discrepancy may reflect differences in experimental context, including the upstream cytokine milieu, cell type composition, disease phase, and sampling time point. Prior studies primarily evaluated lesional human skin or whole-tissue rosacea-like models, in which inflammatory cytokines, barrier disruption, immune-cell infiltration, and neurovascular signals converge to drive a pro-inflammatory STAT3 program. In contrast, our in vitro system examined HaCaT keratinocytes at a defined time point after LL-37 stimulation and subsequent exposure to macrophage-conditioned medium. Thus, the increased pSTAT3 observed after CM_PDLLA_ treatment likely represents an IL-10-linked regulatory STAT3 signal associated with DDIT4 induction and mTORC1 suppression, rather than the pro-inflammatory STAT3/IL-36G axis described in rosacea lesions. Consistent with this context-dependent interpretation, pharmacologic STAT3 inhibition with Stattic reduced the ability of CM_PDLLA_ to suppress pmTOR/mTOR, supporting a requirement for STAT3 activity in this specific macrophage-conditioned regulatory pathway. Likewise, Compound C reversed the CM_PDLLA_-mediated reduction of pmTOR/mTOR, supporting a functional contribution of AMPK to mTORC1 suppression in this model. Therefore, our findings refine, rather than contradict, prior work by suggesting that STAT3 signaling in rosacea-related inflammation may have divergent results depending on the upstream stimulus: inflammatory STAT3 activation may amplify IL-36G-driven inflammation in lesional skin, whereas IL-10-linked STAT3 activation may participate in the DDIT4-associated suppression of mTORC1 under PDLLA-conditioned macrophage signaling.

A previous study of rosacea established that mTORC1 is hyperactivated and participates in a positive feedback loop with cathelicidin/LL-37 [6]. LL-37 can activate mTORC1, reflected by the phosphorylation of mTOR and S6K; activated mTORC1 can further enhance components that facilitate cathelicidin processing and LL-37 amplification, contributing to persistence or relapse [6]. In line with these prior observations, we noted that LL-37 stimulation increased pmTOR and pS6K in keratinocytes, along with the induction of TLR2 and KLK5. Notably, CM_PDLLA_ significantly reduced pmTOR and pS6K; this reduction was accompanied by decreased TLR2 and KLK5 expression levels as well as a decline in LL-37 abundance, indicating functional disruption of the feed-forward program. Given that KLK5 is a critical protease for the processing of cathelicidin into LL-37 peptides [2,3], downregulation of KLK5 provides a plausible biochemical explanation for reduced LL-37 amplification even in an LL-37-primed inflammatory state. Rapamycin, used as an mTORC1 inhibitor control in our model, further suppressed this LL-37–mTORC1 axis, supporting the interpretation that mTORC1 activity is a proximal determinant of the magnitude of LL-37-associated signaling in keratinocytes. Because the inhibitory effects of CM_PDLLA_ were generally weaker than those of rapamycin and were observed in a macrophage-conditioned medium system, PDLLA should not be interpreted as a direct pharmacologic mTORC1 inhibitor. Rather, our results support an indirect immunomodulatory mechanism in which PDLLA-exposed macrophages generate soluble mediators that converge on keratinocyte STAT3/DDIT4, AKT, and AMPK signaling to restrain mTORC1 activity.

This upstream suppression was also evident in vivo. In LL-37-injected inflamed skin, PDLLA reduced pmTOR/mTOR and pS6K/S6K; it concurrently decreased the KLK5, TLR2, and tissue LL-37 levels. These findings are consistent with a model in which PDLLA disrupts the LL-37–mTORC1 positive feedback loop within the tissue microenvironment.

The NLRP3 inflammasome is a cytosolic innate immune signaling platform that classically consists of the sensor (NLRP3), the adaptor (ASC), and pro-caspase-1 [21,22]. Upon assembly, caspase-1 becomes activated and processes pro-inflammatory cytokines into their mature, secreted forms [21,22]. Canonical NLRP3 inflammasome activation is often regarded as a two-step process that comprises priming and activation. Priming, frequently mediated by pattern-recognition receptor signaling and NF-κB activation, increases the transcription of NLRP3 and pro-IL-1β/pro-IL-18 [21,22]. Activation, driven by diverse cellular stress signals (e.g., ionic flux such as K^+^ efflux, mitochondrial dysfunction/reactive oxygen species, and lysosomal destabilization), promotes inflammasome assembly and caspase-1 activation [22].

Importantly for rosacea biology, LL-37 has been shown to drive rosacea-like skin inflammation via NLRP3-dependent caspase-1 activation in experimental systems; inhibition or knockout of NLRP3 attenuates LL-37-induced skin inflammation in mice [4]. Our findings that PDLLA reduces NLRP3, ASC, and caspase-1 cleavage, as well as IL-18 secretion, are thus consistent with a disease-relevant inflammatory pathway. One parsimonious interpretation is that PDLLA suppresses skin inflammation through multiple upstream mechanisms: (i) reduction in LL-37/TLR2/KLK5 amplification and (ii) attenuation of priming and activation signals via the suppression of mTORC1 and NF-κB. Because IL-10–STAT3 signaling can also limit pathological inflammation in other settings [8,23], PDLLA-induced IL-10 may provide an additional layer of inflammasome suppression.

Vascular dysregulation—a central feature of rosacea—includes increased cutaneous blood flow, persistent dilation of blood and lymphatic vessels, increased vascular permeability, and vascular hyperresponsiveness. These changes collectively contribute to hallmark clinical findings such as flushing, persistent erythema, telangiectasia, and tissue swelling [24]. LL-37 is not only an inflammatory peptide but also a vasoactive mediator; it can induce endothelium-dependent vasodilation, increase cutaneous vascular permeability through mast-cell-dependent pathways, and promote angiogenic signaling [25]. Although there is limited direct evidence that LL-37 alone disrupts the vascular basement membrane in human rosacea, chronic LL-37-driven inflammation may indirectly induce basement membrane injury through the upregulation of vascular remodeling mediators and proteases [26]. VEGF is a key mediator of vascular permeability and angiogenesis, and persistent VEGF signaling can destabilize microvascular structure [26]. In the present study, LL-37 increased the VEGF levels and was associated with ultrastructural damage to the vascular basement membrane, whereas PDLLA reduced the VEGF levels and improved ultrastructural integrity.

Current rosacea management is primarily phenotype-based and multimodal, incorporating trigger control, topical and systemic agents, and device-based vascular therapies [16,27]. Existing pharmacologic approaches (e.g., azelaic acid, subantimicrobial-dose doxycycline, and topical ivermectin) have been shown to indirectly modulate the LL-37/TLR2/KLK5 axis by inhibiting protease activity or reducing cathelicidin-related signaling [16,27]. However, these therapies have substantial clinical limitations. They are often not disease-modifying, leading to frequent relapse; they also are hindered by issues such as topical intolerance, concerns about long-term antibiotic stewardship, and the transient nature of symptomatic relief provided by vasoconstrictors or laser-based treatments [16,27].

PDLLA and related polylactic-acid-based injectables are widely used as collagen biostimulators in aesthetic dermatology [28]. Their clinical effect is suspected to involve a controlled, subclinical inflammatory tissue response that leads to collagen remodeling at the injection site and is highly dependent on macrophage–fibroblast interactions [29]. In a prior study, PDLLA injection in aged skin led to increased IL-10 expression and was associated with macrophage-related changes that promoted tissue remodeling, suggesting that PDLLA can modulate the local immune environment beyond volumization [12].

In the context of rosacea-like inflammation, our results support a distinct potential role for PDLLA as a locally delivered immunomodulatory biomaterial that increases IL-10 and downregulates LL-37 amplification and downstream inflammatory pathways, including mTORC1, NF-κB, and the NLRP3 inflammasome. If these mechanistic insights are validated in clinical settings, PDLLA may provide a supportive therapeutic option—in conjunction with existing treatments—by modulating local immune responses. This approach could offer a more sustained regulatory effect than conventional topical agents and may represent an alternative option for patients whose inflammatory symptoms recur despite standard care.

Nevertheless, because PDLLA is an injectable biomaterial, clinical translation for inflammatory facial dermatoses would require a careful safety evaluation. Polylactic-acid-based injectables can cause injection-related adverse events, including inflammatory nodules, foreign-body granulomatous reactions, persistent swelling, and delayed inflammatory responses. Therefore, patient selection, injection plane, dose, particle dispersion, and long-term monitoring should be optimized before PDLLA can be considered for rosacea-related indications.

This study had some limitations. First, the experimental models used here may not fully recapitulate the multifaceted nature of human rosacea. The macrophage and keratinocyte experiments used the THP-1 and HaCaT cell lines, which may differ in baseline TLR2/KLK5/LL-37 and inflammasome responsiveness relative to primary human rosacea lesional keratinocytes and tissue-resident macrophage subsets; validation using primary keratinocytes, primary macrophages, and patient-derived tissues will therefore be required. Second, the in vivo LL-37 injection model reproduces important innate immune features but does not fully capture the chronic, trigger-linked, neurovascular, and microbiome-driven components of human rosacea; we therefore interpret the animal findings as evidence of efficacy in an LL-37-driven rosacea-like inflammation model rather than as a complete representation of human rosacea. Third, although we added a normal control/saline group for the macrophage-polarization analysis and added time-course gross lesion images, the original in vivo experiment did not include a positive drug comparator. Therefore, the therapeutic effect of PDLLA should be interpreted relative to the LL-37/saline inflammatory condition, and future studies should compare PDLLA with standard rosacea therapies or established anti-inflammatory controls. Fourth, although CM_PDLLA_ suppressed mTORC1-related signaling, its inhibitory effects were generally weaker than rapamycin, supporting the interpretation that PDLLA acts through indirect immunomodulation rather than direct mTORC1 inhibition. Fifth, our data support an IL-10-linked association with STAT3/DDIT4, AMPK activation, and mTORC1 suppression, but we did not perform IL-10 neutralization or IL-10 receptor blockade; therefore, IL-10 should be considered a plausible upstream mediator rather than a fully proven causal requirement. The STAT3 and AMPK inhibitor experiments strengthen the downstream pathway analysis, but further work using IL-10 neutralization, IL-10 receptor blockade, STAT3/DDIT4 loss-of-function, and macrophage-depletion or lineage-tracing strategies will be required to map the PDLLA–macrophage–keratinocyte axis more precisely. Finally, this work did not evaluate PDLLA behavior in human rosacea skin under clinically relevant conditions, such as facial skin anatomy, repeated trigger exposure, Demodex or microbial factors, and long-term injection safety. These limitations provide a framework for subsequent translational studies.

## 4. Materials and Methods

### 4.1. Preparation of PDLLA Material

PDLLA used in this study was supplied by VAIM Co., Ltd. (Seoul, Republic of Korea) and corresponds to a commercially available formulation (Juvelook^®^). The material was prepared as sterile injectable microparticles suspended in physiological saline. Particle size distribution was analyzed using a laser diffraction particle size analyzer (Malvern Instruments, UK), revealing a size range of 10–100 μm with a median particle diameter (D50) of 23.4 μm. Prior to experimental use, PDLLA suspensions were thoroughly vortexed to ensure homogeneous dispersion [12,30].

### 4.2. In Vitro Experiments

Human monocytic THP-1 cells and immortalized human keratinocyte HaCaT cells were used for in vitro experiments. All cells were maintained in a humidified incubator at 37 °C with 5% CO_2_.

THP-1 cells were obtained from the Korean Cell Line Bank (KCLB, Seoul, Republic of Korea; KCLB No. 40202) and maintained in RPMI-1640 medium (Gibco, Thermo Fisher Scientific, Waltham, MA, USA) supplemented with 10% fetal bovine serum (Gibco), 1% penicillin–streptomycin (Thermo Fisher Scientific), and 2 mM L-glutamine.

To generate macrophage-like cells, THP-1 monocytes were seeded and differentiated using PMA (Sigma-Aldrich, St. Louis, MO, USA). Cells were treated with 100 nM PMA for 24 h to induce macrophage differentiation. After differentiation, cells were washed twice with PBS to remove residual PMA and incubated in fresh RPMI medium for an additional 24 h to allow for recovery and stabilization of the macrophage-like phenotype.

Differentiated THP-1 macrophages were treated with PBS or PDLLA at the indicated concentrations and incubated for 48 h. CM were collected, centrifuged to remove cellular debris, and stored at −80 °C until further use. Collected CM were used to evaluate cytokine secretion and to stimulate keratinocytes in subsequent experiments.

For macrophage stimulation assays, PDLLA concentrations ranging from 50 to 300 μg/mL were tested to determine concentration-dependent effects on cytokine secretion. Based on preliminary experiments showing the saturation of IL-10 secretion above 200 μg/mL, a PDLLA concentration of 200 μg/mL was used for subsequent experiments.

For the additional dose–response experiments, conditioned media were generated from macrophages treated with 100, 200, or 300 μg/mL PDLLA and then applied to LL-37-stimulated HaCaT cells.

HaCaT keratinocytes were kindly provided by Prof. Jeong-Hee Hong (Gachon University, Incheon, Republic of Korea) and cultured in Dulbecco’s modified Eagle medium (Gibco) containing 10% fetal bovine serum and 1% penicillin–streptomycin.

To generate an inflammatory keratinocyte model, HaCaT cells were seeded and cultured overnight. Inflammatory stimulation was induced by treatment with LL-37 (8 μM; InvivoGen, San Diego, CA, USA) for 24 h [31]. After LL-37 stimulation, cells were treated with macrophage-derived conditioned medium containing PDLLA (CM_PDLLA_) or rapamycin (100 nM; Sigma-Aldrich) as an mTOR inhibitor control. Cells were incubated for an additional 48 h. At the end of the incubation period, CM were collected for cytokine analysis, and cells were harvested for protein analysis.

For STAT3 and AMPK inhibition experiments, LL-37-stimulated HaCaT cells were treated with CM_PDLLA_ and then exposed to Stattic (2.5 μM; Sigma-Aldrich) or Compound C (10 μM; Sigma-Aldrich) for 1 h [32,33].

For in vitro experiments, PDLLA was diluted in serum-free culture medium to the indicated concentrations.

### 4.3. In Vivo Experiments

All animal procedures were conducted in accordance with institutional guidelines and were approved by the Institutional Animal Care and Use Committee of Gachon University (IACUC; LCDI-2025-0065). This study complied with the ethical standards of the Association for Assessment and Accreditation of Laboratory Animal Care International (AAALAC International, Frederick, MD, USA) and adhered to the Animal Research: Reporting of In Vivo Experiments (ARRIVE) guidelines. Each mouse was injected and analyzed individually throughout the study. No humane endpoints were reached during the study.

Female BALB/c mice (7 weeks old) were purchased from Orient Bio (Seongnam, Republic of Korea). Mice were housed under controlled environmental conditions (temperature 22 ± 2 °C, humidity 50–60%) with a 12-h light/dark cycle and free access to food and water.

To induce skin inflammation, LL-37 peptide (320 μM, 40 μL per injection site) was injected into the dorsal skin once daily for 10 consecutive days [34,35,36]. Animals were randomly assigned to experimental groups using a simple randomization method. On day 11, mice received intradermal injections of either saline or PDLLA. PDLLA was suspended in sterile physiological saline at 5 mg/mL; thus, each mouse received 100 μL, corresponding to 0.5 mg PDLLA per mouse. To maintain inflammatory stimulation and prevent spontaneous recovery, LL-37 injections were continued every 2 days thereafter. Skin tissues were collected at designated time points for biochemical and histologic analyses. Gross lesion images were additionally documented at day 11, day 25, and day 39 to evaluate time-dependent changes after a single PDLLA injection.

### 4.4. ELISA

Total protein extracts were prepared using RIPA lysis buffer (ATTO Corporation, Tokyo, Japan) containing protease and phosphatase inhibitor cocktails (Roche Diagnostics, Mannheim, Germany). Protein concentrations were measured using a bicinchoninic acid assay kit (Thermo Fisher Scientific).

Ninety-six-well high-binding microplates (Corning Inc., Corning, NY, USA) were coated overnight at 4 °C with sample diluted in coating buffer (0.05 M carbonate–bicarbonate buffer, pH 9.6). After coating, plates were washed three times with PBS containing 0.1% Tween-20. To block nonspecific binding, plates were incubated with blocking buffer consisting of 1% bovine serum albumin in PBS for 1 h at room temperature. Plates were washed three times with PBS containing Tween-20 to remove unbound proteins. Primary antibodies (Table S1) were added to each well and incubated for 1 h at room temperature. After the wells had been washed, horseradish-peroxidase-conjugated secondary antibodies were added and incubated for 2 h. Color development was achieved using tetramethylbenzidine substrate solution (Sigma-Aldrich), and the reaction was terminated with 2 N sulfuric acid. Absorbance was measured at 450 nm. Background-subtracted absorbance values were normalized to those of the corresponding control group, which was assigned a value of 1. All biological samples were analyzed in technical triplicate.

### 4.5. Western Blotting

Equal amounts of protein (20–40 μg) were separated by sodium dodecyl sulfate–polyacrylamide gel electrophoresis and transferred onto polyvinylidene fluoride membranes (Millipore, Burlington, MA, USA). Membranes were blocked with 5% skim milk in Tris-buffered saline containing 0.1% Tween-20 for 1 h at room temperature. Membranes were incubated overnight at 4 °C with primary antibodies (Table S1), washed with Tris-buffered saline containing Tween-20, and then incubated with horseradish-peroxidase-conjugated secondary antibodies for 1 h at room temperature. Protein bands were detected using an enhanced chemiluminescence detection system (Cytiva) and visualized with a ChemiDoc imaging system (Bio-Rad, Hercules, CA, USA). Band intensities were quantified using ImageJ software (version 1.53s; National Institutes of Health, Bethesda, MD, USA) and normalized to β-actin.

### 4.6. Immunocytochemistry

For immunocytochemical analysis of NF-κB localization, HaCaT cells were cultured in eight-well chambers. Cells were fixed with 4% paraformaldehyde for 15 min and permeabilized with 0.1% Triton X-100 for 10 min. After they had been blocked with 1% normal serum, cells were incubated overnight at 4 °C with an NF-κB antibody (Table S1). Cells were then incubated with fluorescent secondary antibodies for 1 h at room temperature. Nuclei were counterstained with 4′,6-diamidino-2-phenylindole (DAPI). Fluorescence images were obtained using an LSM-710 confocal microscope (Carl Zeiss, Oberkochen, Germany) at the core facility for cell-to-in vivo imaging. In each experimental group, five randomly selected fields per section were analyzed and quantified using ZEN imaging software (version 5.1; Carl Zeiss).

### 4.7. Immunohistochemistry

Skin tissue samples collected from mice were fixed in 4% paraformaldehyde for 72 h at 4 °C and subsequently embedded in paraffin. Paraffin blocks were sectioned at a thickness of 7 μm using a microtome and mounted on slides. Sections were deparaffinized in xylene and rehydrated through a graded ethanol series (100%, 95%, 80%, and 70%), then rinsed in distilled water. After tissues had been washed with PBS, they were permeabilized with 0.1% Triton X-100 in PBS for 10 min. To reduce nonspecific binding, sections were blocked with 1% normal serum for 1 h at room temperature. Sections were then incubated overnight at 4 °C with an NF-κB antibody (Table S1) diluted in blocking buffer. After this incubation, sections were washed three times in PBS, incubated with biotinylated secondary antibodies (Vector Laboratories, Burlingame, CA, USA) for 1 h at room temperature, and incubated ABC kit (Vector Laboratories) for 30 min. After section were treated with a DAB solution (Sigma-Aldrich) for 5 min. Nuclei were counterstained with hematoxylin for 1 min. After a final washing step, slides were mounted using DPX mounting solution (Sigma-Aldrich). Stained tissues were scanned using a slide scanner (VS200; Olympus Corporation, Tokyo, Japan), and 10 images were randomly captured for analysis.

### 4.8. Hematoxylin and Eosin

Skin tissues were deparaffinized in xylene and rehydrated through a graded ethanol series (100%, 95%, 80%, and 70%), then rinsed in distilled water. Deparaffinized slides were stained with hematoxylin (KPNT; Cheongju, Republic of Korea), rinsed, and then counterstained with eosin (KPNT). Slides were dehydrated, mounted, and examined under a microscope. Stained tissues were scanned using a slide scanner (VS200; Olympus Corporation), and 10 images were randomly captured for analysis.

### 4.9. Transmission Electron Microscopy

Skin tissues were fixed in 2% paraformaldehyde and 2% glutaraldehyde, followed by post-fixation in osmium tetroxide. Samples were dehydrated through a graded ethanol series, embedded in epoxy resin, and ultrathin sections (70–90 nm) were prepared. Sections were stained with uranyl acetate and lead citrate, and examined using a transmission electron microscope (HT7800; Hitachi, Tokyo, Japan).

### 4.10. Statistical Analysis

Sample size was determined based on previous studies using similar experimental models and practical considerations. Outcome assessment and data analysis were performed in a blinded manner where possible. All experiments were performed using at least three independent biological replicates. Data are expressed as mean ± standard deviation. Statistical analysis was performed using SPSS version 26 (IBM Corp., Armonk, NY, USA). Although the data distribution was assessed using the Shapiro–Wilk test, the sample size in each group was insufficient to reliably evaluate normality or homogeneity of variance. Accordingly, nonparametric statistical methods were used for all analyses. The Kruskal–Wallis test was applied, followed by pairwise comparisons via the Mann–Whitney U test. Statistical significance and the number of biological replicates are indicated in the figure legends.

## 5. Conclusions

PDLLA increased IL-10 secretion from macrophages, and through macrophage-conditioned crosstalk, attenuated LL-37-induced inflammatory signaling in HaCaT keratinocytes and in an intradermal LL-37 rosacea-like mouse model. CM_PDLLA_ restored pSTAT3/DDIT4 signaling, reduced AKT phosphorylation, increased AMPK activation, and suppressed mTORC1 signaling (pmTOR/pS6K), which was associated with downregulation of the TLR2/KLK5/LL-37 amplification axis and reduced NF-κB activation, VEGF production, and NLRP3 inflammasome/IL-18 responses. Additional pharmacologic inhibition experiments further supported the functional involvement of STAT3 and AMPK in CM_PDLLA_-mediated mTORC1 suppression, although these findings do not imply that global STAT3 activation is uniformly protective in rosacea. In vivo, PDLLA reduced inflammatory infiltration, improved vascular basement membrane ultrastructure, and decreased gross lesion severity. Overall, PDLLA may represent a locally delivered, indirect immunomodulatory approach targeting the LL-37–mTORC1 amplification circuit in rosacea-like inflammation, rather than a direct mTORC1 inhibitor. Further studies using IL-10 neutralization, primary human cells, chronic/neurovascular rosacea models with standard therapeutic comparators, and safety assessments of injectable PDLLA are required before clinical translation.

## Data Availability

All data are contained within this article.

## Supplementary Materials

The following supporting information can be downloaded at: https://www.mdpi.com/article/10.3390/ijms27146425/s1.
