# Supplementary material for: Immunomodulatory Effects of Poly-D,L-Lactic Acid on LL-37-Driven Rosacea-like Inflammation via Suppression of mTORC1 Signaling
Source: Int J Mol Sci. 2026 Jul 19;27(14):6425. doi: 10.3390/ijms27146425 (PMC13410482; doi:10.3390/ijms27146425)
Supplement: Supplementary file 1 [file ijms-27-06425-s001.zip › ijms-4304301-supplementary.pdf]

International Journal of Molecular Sciences

Article

Immunomodulatory Effects of Poly-D,L-Lactic Acid on LL-37-Driven Rosacea-like Inflammation via Suppression of mTORC1 Signaling

Kyung-A Byun [1,2,3,†], Jeyoung Park [4,†], Seyeon Oh [2,3], Jiyeon Shin [5], Suk Bae Seo [6], Kuk Hui Son [7,*], and Kyunghee Byun [1,3,8,*]

1 Department of Anatomy & Cell Biology, College of Medicine, Gachon University, Incheon 21936, Republic of Korea
2 LIBON Inc., Incheon 22006, Republic of Korea
3 Functional Cellular Networks Laboratory, Lee Gil Ya Cancer and Diabetes Institute, Gachon University, Incheon 21999, Republic of Korea
4 Oracle Dermatology Clinic, Seoul 06097, Republic of Korea
5 Heve Dermatology Clinic, Seoul 06035, Republic of Korea
6 SeoAh Song Dermatologic Clinic, Seoul 05557, Republic of Korea
7 Department of Thoracic and Cardiovascular Surgery, Gachon University Gil Medical Center, Gachon University, Incheon 21565, Republic of Korea
8 Department of Health Sciences and Technology, Gachon Advanced Institute for Health & Sciences and Technology (GAIHST), Gachon University, Incheon 21999, Republic of Korea
* Correspondence: dr632@gachon.ac.kr (K.H.S.); khbyun1@gachon.ac.kr (K.B.); Tel.: +82-32-460-3666 (K.H.S.); +82-32-899-6511 (K.B.)
† These authors contributed equally to this work.

 https://doi.org/10.3390/ijms27146425

Table S1. This is a table. Tables should be placed in the main text near to the first time they are cited.

Antibody	Company	Dilution rate		
		Western blot	ELISA	ICC/IHC
β-actin	Cell Signaling Technology	1:1,000	-	-
STAT3	Cell Signaling Technology	1:1,000	-	-
pSTAT3	HUABIO	1:1,000	-	-
DDIT4	Cell Signaling Technology	1:1,000	-	-
AKT	BD biosciences	1:1,000	-	-
pAKT	Cell Signaling Technology	1:1,000	-	-
AMPK	Affinity	1:1,000	-	-
pAMPK	Cell Signaling Technology	1:1,000	-	-
mTOR	LSBIO	1:1,000	-	-
pmTOR	Abbexa	1:1,000	-	-
S6K	Cell Signaling Technology	1:1,000	-	-
pS6K	Cell Signaling Technology	1:1,000	-	-
KLK5	Abcam	1:1,000	-	-
TLR2	Novus Biologicals	1:1,000	-	-
LL-37	Santa Cruz	-	1:500	-
NF-κB	Cell Signaling Technology	-	-	1:100
VEGF	Fine Test	-	1:500	-
NLRP3	BOSTER	1:1,000	-	-
ASC	Santa Cruz Biotechnology	1:1,000	-	-
Caspase-1	Santa Cruz Biotechnology	1:1,000	-	-
IL-18	Invitrogen	-	1:500	-
CD86	Novus Biologicals	1:1,000	-	-
CD206	Novus Biologicals	1:1,000	-	-
IL-10	Fine Test	-	1:500	-

https://doi.org/10.3390/xxxxx

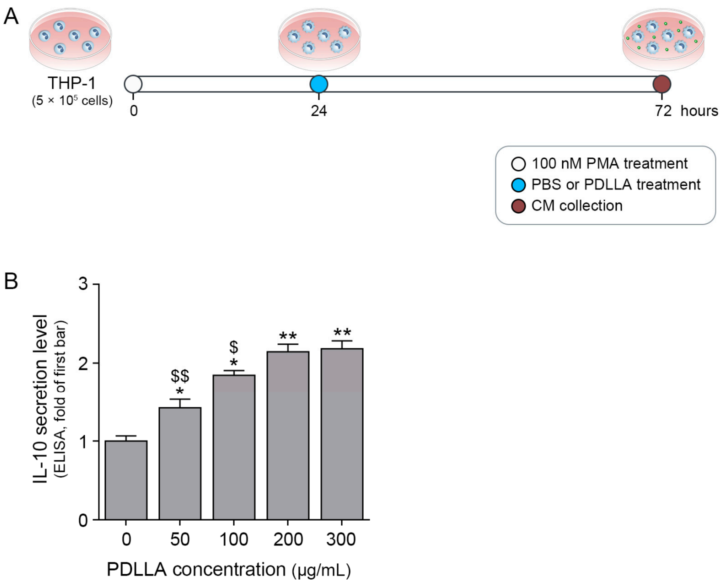

Figure S1. PDLLA induces IL-10 secretion in LL-37-stimulated macrophages. (A) Experimental schematic for PDLLA treatment in macrophages. THP-1 monocytes were seeded and differentiated into macrophage-like cells by treatment with 100 nM phorbol 12-myristate 13-acetate (PMA) for 24 h. After differentiation, cells were treated with phosphate-buffered saline (PBS) or PDLLA at the indicated concentrations. Conditioned media (CM) were collected after 48 h. (B) Concentration-dependent changes in IL-10 secretion after PDLLA treatment were measured by the enzyme-linked immunosorbent assay (ELISA) method. IL-10 secretion increased in a dose-dependent manner and reached saturation at concentrations above 200 μg/mL PDLLA. Data are presented as mean ± standard deviation from biological replicates. Data are presented as mean ± SD (n = 5 independent biological replicates). **, $p < 0.01$, vs. first bar; $, $p < 0.05$ and $$, $p < 0.01$, vs. second bar; #, $p < 0.05$, vs. third bar.

https://doi.org/10.3390/xxxxx

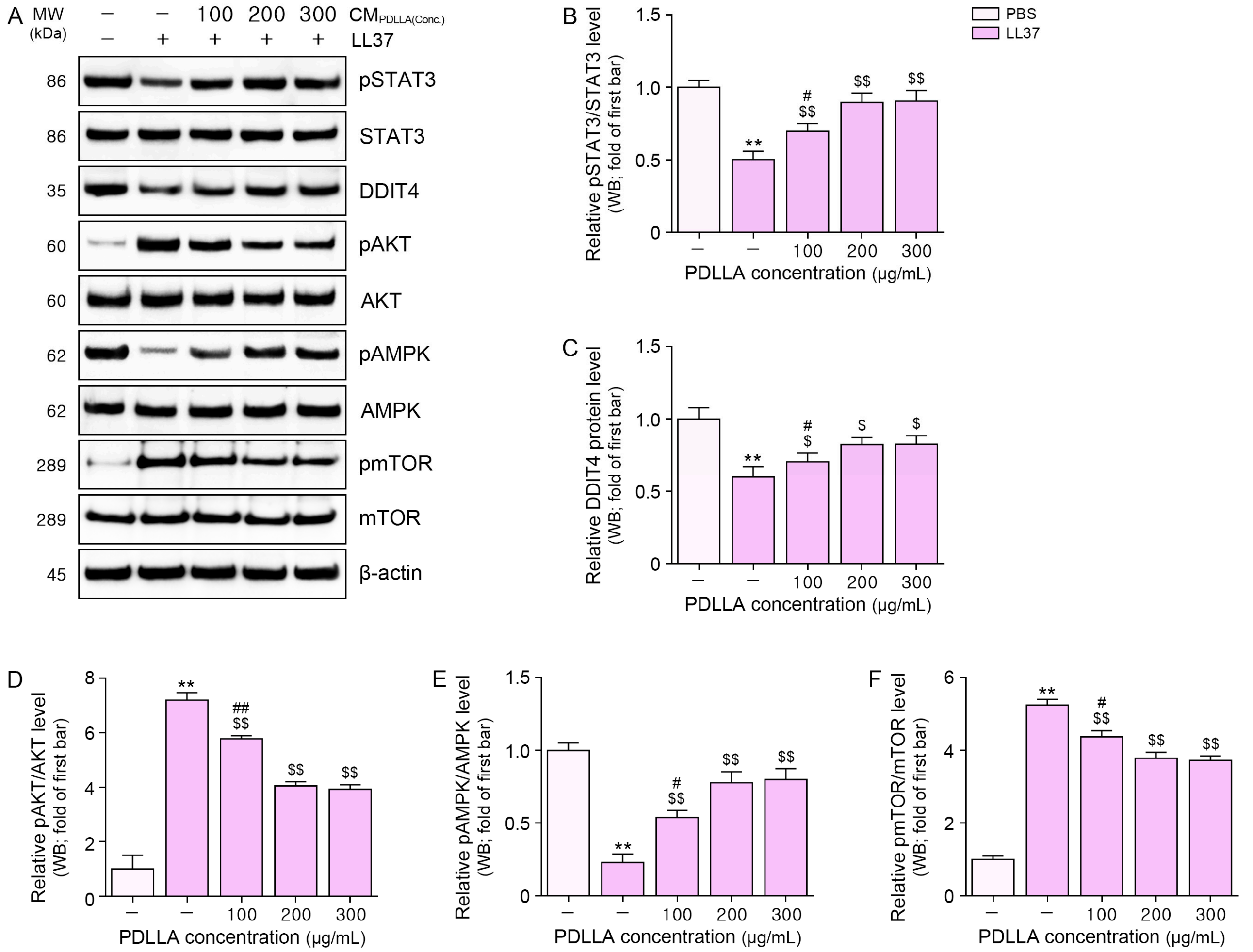

Figure S2. PDLLA-conditioned medium dose-dependently restores mTOR signaling in LL-37-treated keratinocytes. (A) Western blot analysis of mTOR-related signaling molecules, including phosphorylated STAT3 (pSTAT3 Tyr705), DDIT4, phosphorylated AKT (pAKT Ser473), phosphorylated AMPK (pAMPK Thr172), and phosphorylated mTOR (pmTOR Ser2448). (B–F) Quantification of western blot band intensities normalized to total protein levels. Data are presented as mean ± SD (n = 5 independent biological replicates). **, $p < 0.01$, vs. first bar; $, $p < 0.05$ and $$, $p < 0.01$, vs. second bar; #, $p < 0.05$ and ##, $p < 0.01$, vs. fourth bar. AMPK, AMP-activated protein kinase; DDIT4, DNA-damage-inducible transcript 4; mTOR, mechanistic Target of Rapamycin; PDLLA, poly-D,L-lactic acid; SD, standard deviation; STAT3, Signal Transducer and Activator of Transcription 3.

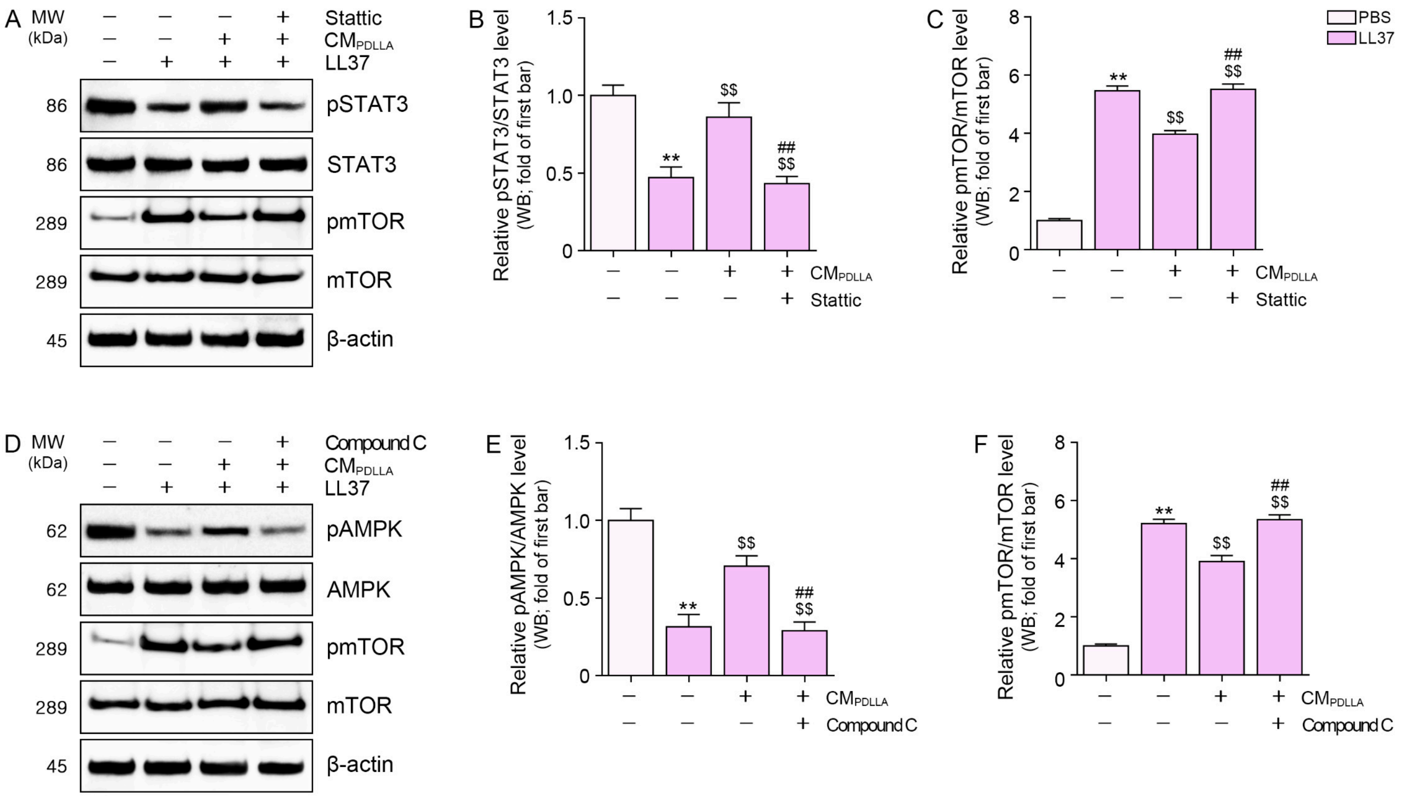

Figure S3. Inhibition of STAT3 or AMPK reverses the suppressive effect of PDLLA-conditioned medium on mTOR signaling in LL-37-treated keratinocytes. (A) Western blot analysis of phosphorylated STAT3 (pSTAT3 Tyr705) and phosphorylated mTOR (pmTOR Ser2448) in the presence or absence of CMPDLLA and the Stattic. (B,C) Quantification of western blot band intensities normalized to total protein levels. (D) Western blot analysis of phosphorylated AMPK (pAMPK Thr172) and phosphorylated mTOR (pmTOR Ser2448) in the presence or absence of CMPDLLA and the Compound C. (E,F) Quantification of western blot band intensities normalized to total protein levels. Data are presented as mean ± SD ($n = 5$ independent biological replicates). **, $p < 0.01$, vs. first bar; $$, $p < 0.01$, vs. second bar; ##, $p < 0.01$, vs. third bar. AMPK, AMP-activated protein kinase; mTOR, mechanistic Target of Rapamycin; PDLLA, poly-D,L-lactic acid; SD, standard deviation; STAT3, Signal Transducer and Activator of Transcription 3.

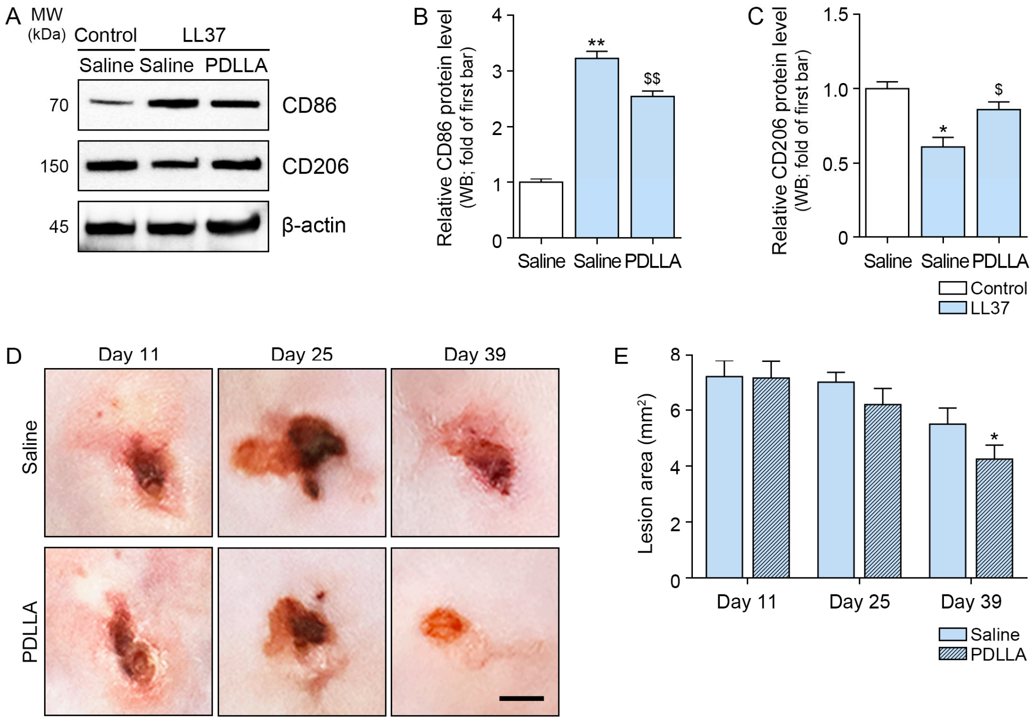

Figure S4. PDLLA modulates macrophage polarization and attenuates lesion progression in LL-37-induced skin inflammation. (A) Western blot analysis of M1 macrophage marker CD86 and M2 macrophage marker CD206. (B,C) Quantification of western blot band intensities normalized to total protein levels. (D,E) Representative clinical photographs of skin lesions at Days 11, 25, and 39 following saline or PDLLA treatment. Scale bar = 2 mm. Data are presented as mean ± SD (n = 5 mice per group). *, $p < 0.05$ and **, $p < 0.01$, vs. Saline. PDLLA, poly-D,L-lactic acid; SD, standard deviation.

https://doi.org/10.3390/xxxxx
